# Microvesicle release from inner segments of healthy photoreceptors is a conserved phenomenon in mammalian species

**DOI:** 10.1242/dmm.049871

**Published:** 2022-11-23

**Authors:** Tylor R. Lewis, Sebastien Phan, Keun-Young Kim, Isha Jha, Carson M. Castillo, Jin-Dong Ding, Benjamin S. Sajdak, Dana K. Merriman, Mark H. Ellisman, Vadim Y. Arshavsky

**Affiliations:** ^1^ Department of Ophthalmology, Duke University Medical Center, Durham, NC 27710, USA; ^2^ National Center for Microscopy and Imaging Research, School of Medicine, University of California San Diego, La Jolla, CA 92093, USA; ^3^ Department of Biology, University of Wisconsin Oshkosh, Oshkosh, WI 54901, USA; ^4^ Fauna Bio Inc., Emeryville, CA 94608, USA; ^5^ Department of Pharmacology and Cancer Biology, Duke University Medical Center, Durham, NC 27710, USA

**Keywords:** Microvesicle, Mitochondria, Photoreceptor, Retina, Rhodopsin, Vision

## Abstract

Many inherited visual diseases arise from mutations that affect the structure and function of photoreceptor cells. In some cases, the pathology is accompanied by a massive release of extracellular vesicles from affected photoreceptors. In this study, we addressed whether vesicular release is an exclusive response to ongoing pathology or a normal homeostatic phenomenon amplified in disease. We analyzed the ultrastructure of normal photoreceptors from both rod- and cone-dominant mammalian species and found that these cells release microvesicles budding from their inner segment compartment. Inner segment-derived microvesicles vary in their content, with some of them containing the visual pigment rhodopsin and others appearing to be interconnected with mitochondria. These data suggest the existence of a fundamental process whereby healthy mammalian photoreceptors release mistrafficked or damaged inner segment material as microvesicles into the interphotoreceptor space. This release may be greatly enhanced under pathological conditions associated with defects in protein targeting and trafficking.

This article has an associated First Person interview with the first author of the paper.

## Teaser

**Summary:** Healthy photoreceptor cells release extracellular vesicles directly from their inner segment compartment. This process appears to be amplified in some photoreceptor degenerative models for disposal of mistrafficked proteins.

## INTRODUCTION

TheFig. S1 rod and cone photoreceptor cells of the vertebrate retina perform the first steps in vision by capturing light and generating an electric signal ultimately transmitted to the brain. Photoreceptors are highly specialized to perform this function, and this specialization is reflected in their compartmentalized organization (reviewed in Pearring et al., 2013; Goldberg et al., 2016; Wensel et al., 2016; Spencer et al., 2020). Visual signaling takes place in the ciliary outer segment, while most housekeeping functions are performed by the adjacent inner segment. On the other end of the cell resides the synaptic terminal, which conveys the visual response to other retinal neurons.

The exquisite structural organization of photoreceptor cells can be distorted by a variety of mutations associated with inherited retinal diseases. A sizable subset of these mutations causes a massive accumulation of extracellular vesicles in the space surrounding mutant cells. In some cases, these vesicles originate from the outer segment as a result of defects in its formation, such as in *rds* and *Prcd* knockout mice (Salinas et al., 2017; Spencer et al., 2019). In many other cases, extracellular vesicles appear to be released from the inner segment, such as in *pcd* mice (Blanks et al., 1982; Blanks and Spee, 1992), *tubby* mice (Heckenlively et al., 1995), *Tulp1* knockout mice (Hagstrom et al., 1999), *Ift88* hypomorphic mutant mice (Pazour et al., 2002), *Ift172* conditional knockout mice (Gupta et al., 2018) and *Bbs8* conditional knockout mice (Dilan et al., 2018). Intriguingly, each of the proteins mutated in the latter group is implicated in supporting protein trafficking, including the trafficking of the visual pigment rhodopsin from the inner to the outer segment. A comparable vesicular accumulation around inner segments takes place in mice, rabbits, rats and frogs transgenically expressing various rhodopsin mutants (Li et al., 1996; Kondo et al., 2009; Concepcion and Chen, 2010; Lodowski et al., 2013; LaVail et al., 2018; Ropelewski and Imanishi, 2020). It has been suggested that, at least in the case of mutant rhodopsin, this vesicular release serves as a safeguard mechanism counteracting the pathological accumulation of mislocalized protein in the inner segment (Lodowski et al., 2013). What remained unknown is whether this phenomenon occurs exclusively under pathological conditions or it is a normal homeostatic process amplified in mutant photoreceptors to counteract the ongoing pathology.

The goal of our study was to determine whether healthy mammalian photoreceptor cells are able to release extracellular vesicles from their inner segments. We have found that retinas of wild-type (WT) mice, rats and 13-lined ground squirrels (13LGS) occasionally contain extracellular vesicles located next to photoreceptor inner segments. Using 3D electron tomography (3D-ET), which provides resolution on the nanometer scale, we observed vesicles budding directly from the photoreceptor inner segment plasma membrane, which qualifies them as microvesicles. These microvesicles vary in their content, with some containing rhodopsin and others appearing to be interconnected with inner segment mitochondria. These data show that healthy WT photoreceptors release microvesicles into the interphotoreceptor space and suggest that the massive release of extracellular vesicles in pathology represents an amplification of this normal homeostatic process.

## RESULTS

### Microvesicle release from the inner segments of WT mouse photoreceptors

**Fig. 1.**
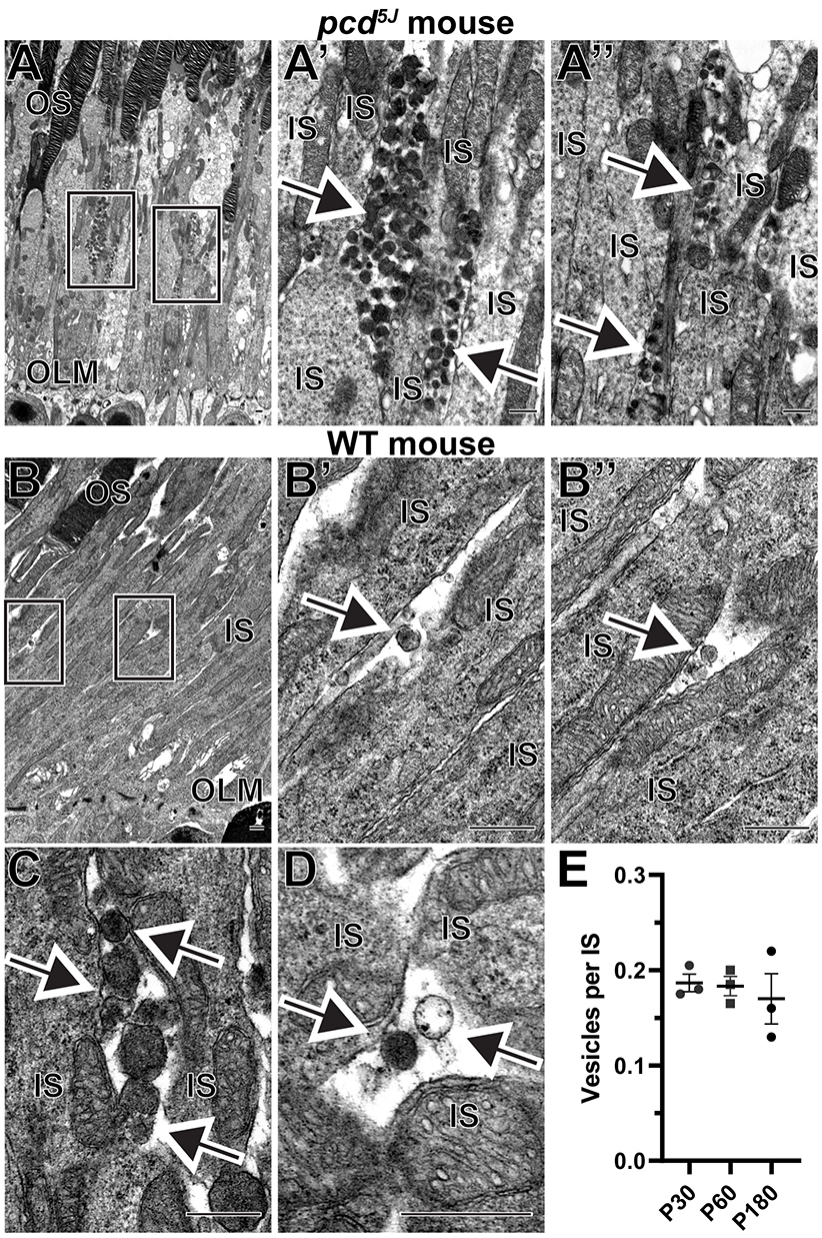
**Extracellular vesicles are located next to inner segments of wild-type (WT) and *pcd^5J^* mouse photoreceptors.** (A) Representative transmission electron microscopy (TEM) images of retinal sections from 22-day-old homozygous *pcd^5J^* mice stained with osmium tetroxide. Boxed areas in A are magnified in A′ and A″. (B) A low-magnification TEM image of a WT mouse retinal section stained with osmium tetroxide. Boxed areas are magnified in B′ and B″. (C) An example of a cluster containing several vesicles. (D) An example of two neighboring vesicles with different staining densities. (E) The frequency of vesicles located next to photoreceptor inner segments in WT mice at P30, P60 and P180. The data are expressed as the number of vesicles per inner segment within a region of the 70 nm-thick retinal section containing 200 photoreceptors. Each data point represents an individual mouse; the bars represent mean±s.e.m. One-way ANOVA test revealed no statistically significant differences across ages (*P*=0.7775). In all panels, arrows point to extracellular vesicles. IS, inner segment; OLM, outer limiting membrane; OS, outer segment. Scale bars: 0.5 µm. A total of four *pcd^5J^* and nine WT mice were analyzed.

Before analyzing WT retinas, we revisited the phenotype of the *pcd* mouse as a benchmark for significant extracellular vesicle accumulation around photoreceptor inner segments (Blanks et al., 1982; Blanks and Spee, 1992). *pcd* mice bear various mutations in the *Agtpbp1* gene, also known as *Nna1* (Fernandez-Gonzalez et al., 2002). This gene encodes a deglutamylase that regulates microtubule modifications in the photoreceptor connecting cilium (Bosch Grau et al., 2017) and other neurons (Rogowski et al., 2010). We used transmission electron microscopy (TEM) to examine the retinal ultrastructure of the homozygous *pcd^5J^* line at an age preceding major photoreceptor cell loss in this model. As previously reported for other *pcd* lines, we observed a massive accumulation of extracellular vesicles specifically in the space between photoreceptor inner segments (Fig. 1A-A″).

We next analyzed the ultrastructure of WT mouse retinas and identified the presence of small numbers of extracellular vesicles next to photoreceptor inner segments with shape and size comparable to those of the vesicles accumulating in *pcd^5J^* mice (Fig. 1B-B″). In most cases, we observed individual vesicles; however, there were instances of pockets containing multiple vesicles (Fig. 1C). Interestingly, the staining density of these vesicles varied. Although the majority were darkly stained, a small number were stained very lightly, indicating variability in their contents (Fig. 1D). The diameter of these vesicles typically ranged between 100 nm and 200 nm. We assessed the frequency at which these vesicles are observed in WT mice and found that there was approximately one vesicle for every five to six photoreceptor inner segments in the 70 nm-thick retinal section (Fig. 1E). Considering the average vesicle diameter of ∼150 nm (which roughly corresponds to two sections) and the average inner segment diameter of ∼1.5 µm (which roughly corresponds to 20 sections), we estimate that around two vesicles are located next to each photoreceptor inner segment. We conducted this analysis at three different mouse ages – postnatal day (P)30, P60 and P180 – and found no significant differences, at least within this age range.

Considering the nature of these vesicles, there are two types of extracellular vesicles released from cells under normal physiological conditions: exosomes and microvesicles (Raposo and Stoorvogel, 2013). Exosomes are released as a large cluster upon fusion of a multivesicular body with the plasma membrane. They are typically less than 100 nm in diameter. In contrast, microvesicles are released individually via direct budding from the plasma membrane. They are typically larger, between 100 nm and 1000 nm in diameter. Given that the majority of vesicles that we observed are singular and over 100 nm in diameter, they are likely to be microvesicles.

**Fig. 2.**
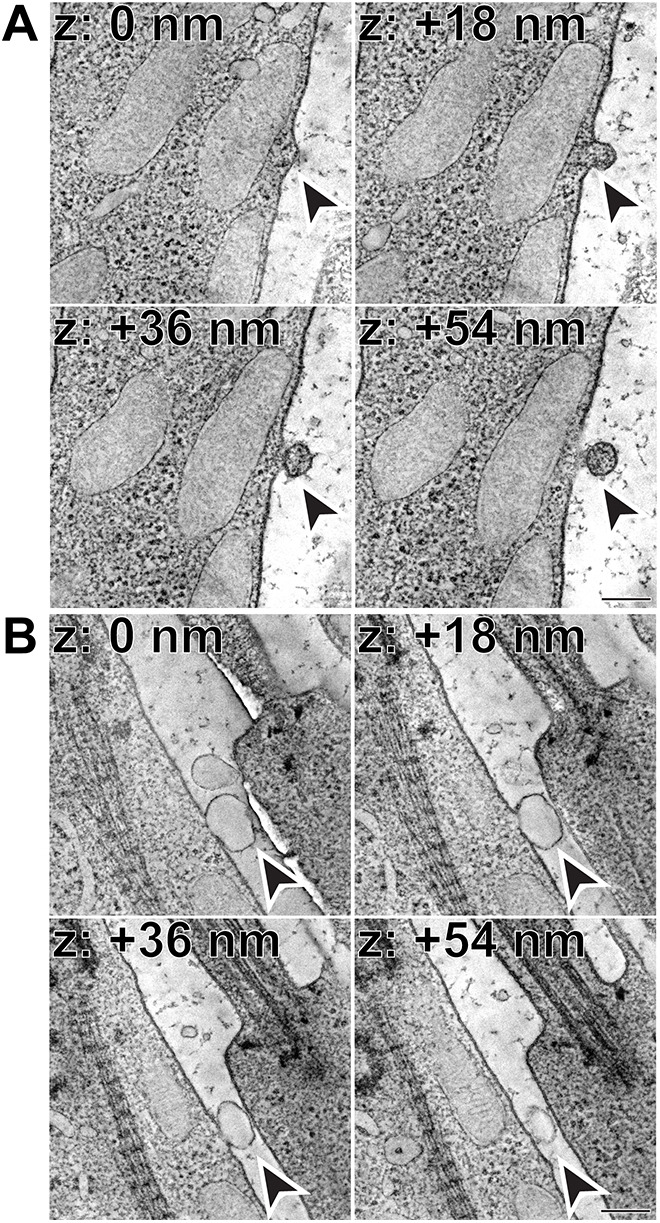
**Microvesicles released from the inner segment plasma membrane.** (A) Representative *z*-sections at the depths of 0, +18, +36 and +54 nm obtained from a 3D electron tomogram of a 250 nm-thick WT mouse retinal section stained with tannic acid/uranyl acetate. Full tomogram is shown in Movie 1. Arrowheads depict a microvesicle with a staining pattern matching that of the cytoplasm in the process of being released. Tomogram pixel size is 1.4 nm; scale bar: 0.2 µm. (B) Representative *z*-sections at the depths of 0, +18, +36 and +54 nm obtained from a 3D electron tomogram of a 250 nm-thick WT mouse retinal section stained with tannic acid/uranyl acetate. Full tomogram is shown in Movie 2. Arrowheads depict a clear microvesicle in the process of being released. Tomogram pixel size is 1.4 nm; scale bar: 0.2 µm. The data are selected from a total of six tomograms documenting microvesicle release from the plasma membrane obtained from three WT mice.

To determine whether these vesicles are indeed microvesicles that originate from the inner segment plasma membrane, we used 3D-ET. Compared to conventional TEM, for which *z*-resolution is limited by the thickness of the tissue section (typically ∼70 nm), 3D-ET provides a *z*-resolution of ∼1 nm, which enabled us to precisely follow the 3D membrane architecture of vesicles in the process of their release. The examples in Fig. 2 and Movies 1 and 2 illustrate outwardly budding vesicles in which the membrane is still continuous with the plasma membrane of the inner segment. Although this was a relatively rare phenomenon to capture at the exact moment of vesicle budding, these tomograms provide compelling evidence that the extracellular vesicles in question are microvesicles released from the inner segments of normal photoreceptors.

As in the TEM images, the content of these extracellular vesicles varied. In some cases, vesicles were filled with material matching that of the inner segment cytoplasm (Fig. 2A), whereas in others, vesicles appeared ‘empty’ (Fig. 2B).

### Some extracellular vesicular structures are connected to inner segment mitochondria

**Fig. 3.**
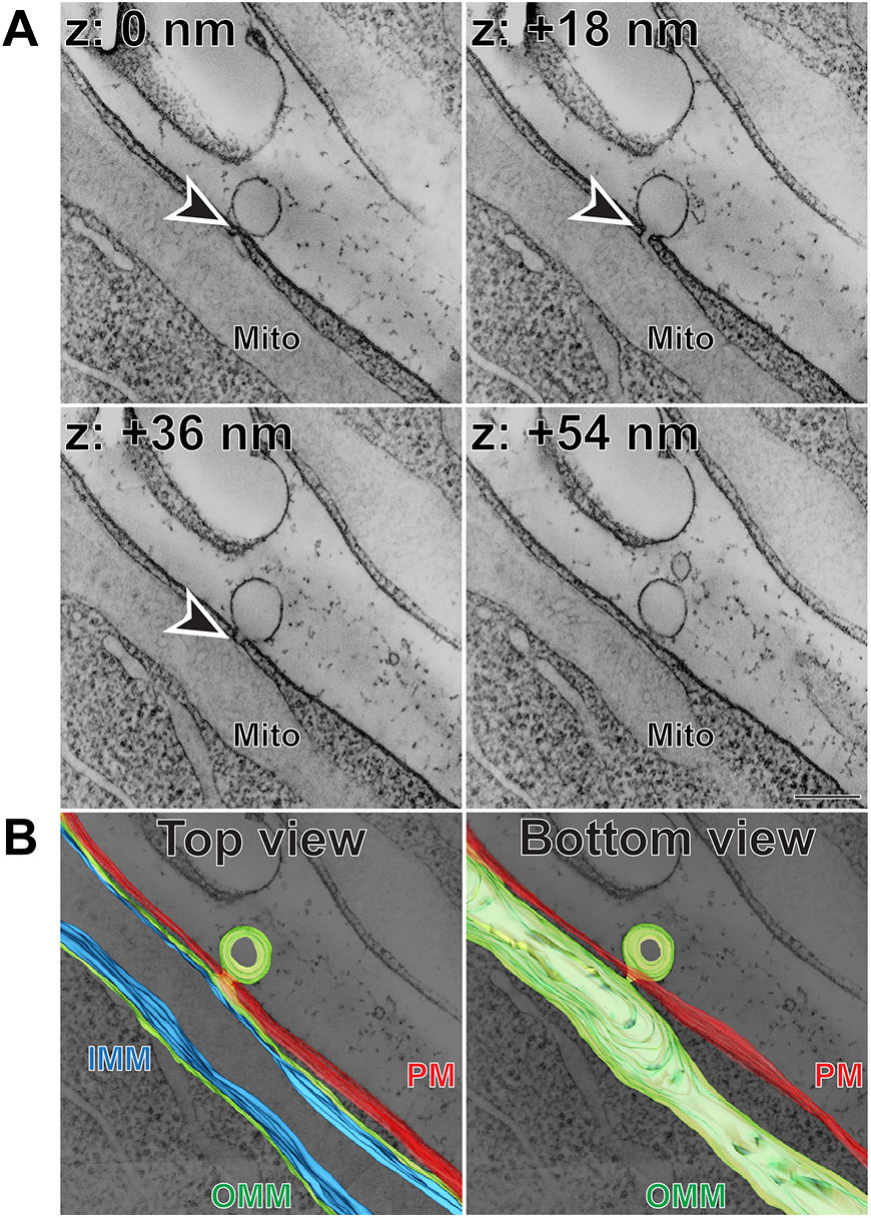
**Extracellular vesicular structure connected to inner segment mitochondrion through a short membrane tunnel.** (A) Representative *z*-sections at the depths of 0, +18, +36 and +54 nm obtained from a 3D electron tomogram of a 750 nm-thick WT mouse retinal section stained with tannic acid/uranyl acetate. Full tomogram is shown in Movie 3. Arrowheads depict a membrane tunnel connecting the budding vesicle with an inner segment mitochondrion. Tomogram pixel size is 1.5 nm; scale bar: 0.2 µm. (B) The corresponding 3D segmentation in two views: from the top as in A and from the bottom. Full segmentation is shown in Movie 5. IMM, inner mitochondrial membrane (blue); Mito, mitochondrion; OMM, outer mitochondrial membrane (green); PM, plasma membrane (red). The data are selected from a total of 12 tomograms documenting extracellular vesicular structures connected to mitochondrial membranes obtained from three WT mice.

**Fig. 4.**
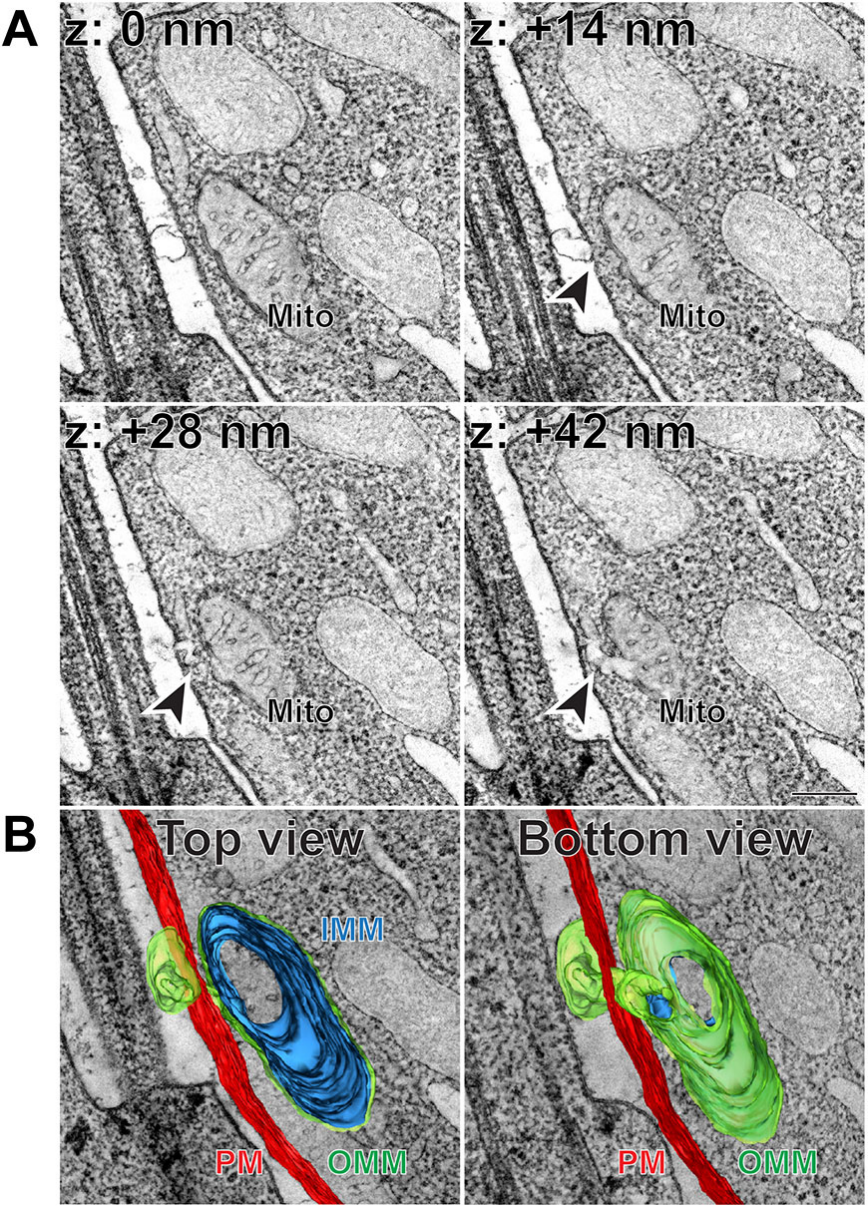
**Extracellular vesicular structure connected to inner segment mitochondrion through a complex membrane tunnel.** (A) Representative *z*-sections at the depths of 0, +14, +28 and +42 nm obtained from a 3D electron tomogram of a 250 nm-thick WT mouse retinal section stained with tannic acid/uranyl acetate. Full tomogram is shown in Movie 4. Arrowheads depict a membrane tunnel connecting the budding vesicle with an inner segment mitochondrion. Tomogram pixel size is 1.4 nm; scale bar: 0.2 µm. (B) The corresponding 3D segmentation in two views: from the top as in A and from the bottom. Full segmentation is shown in Movie 6. IMM, inner mitochondrial membrane (blue); Mito, mitochondrion; OMM, outer mitochondrial membrane (green); PM, plasma membrane (red). The data are selected from a total of 12 tomograms documenting extracellular vesicular structures connected to mitochondrial membranes obtained from three WT mice.

The 3D-ET also revealed several examples of rather complex structures in which a budding vesicle is connected to the outer mitochondrial membrane through a membrane tunnel crossing the inner segment plasma membrane (Fig. 3A, Fig. 4A; Movies 3 and 4). The appearance of the connecting tunnels varied in length and complexity, which is particularly easy to appreciate in segmented images (Fig. 3B, Fig. 4B; Movies 5 and 6). The content of the extracellular vesicular component of these structures was rather clear, and they did not contain any membranes inside.

This result is intriguing in the context of a recent study performed with WT zebrafish cone photoreceptors (Giarmarco et al., 2020). The authors observed electron-dense structures associated with mitochondria in the inner segment. Some of these structures were elongated into tubules, which sometimes crossed the inner segment plasma membrane and formed extracellular networks. A comparable observation of electron-dense material extruding outside the cell had been reported earlier in another fish species, walleye (Januschka et al., 1987), as well as more recently in primates (Hayes et al., 2021). However, there are two distinct differences between their and our observations. First, the staining density of all mitochondria-associated structures in previous reports, including the extracellular extensions, was consistently electron dense. In contrast, all mitochondria-derived structures that we observed in mice were lightly stained. Second, the extracellular components of the structures that we observed had a vesicular profile, whereas at least some of those observed in zebrafish were tubular. Perhaps at least some of these differences may be attributed not only to the species studied, but also to the type of photoreceptor analyzed. In the zebrafish study, mitochondrial extrusion occurred mostly in cones (Giarmarco et al., 2020), whereas our observations were made in rods.

### Microvesicle release is conserved in rod- and cone-dominant species

**Fig. 5.**
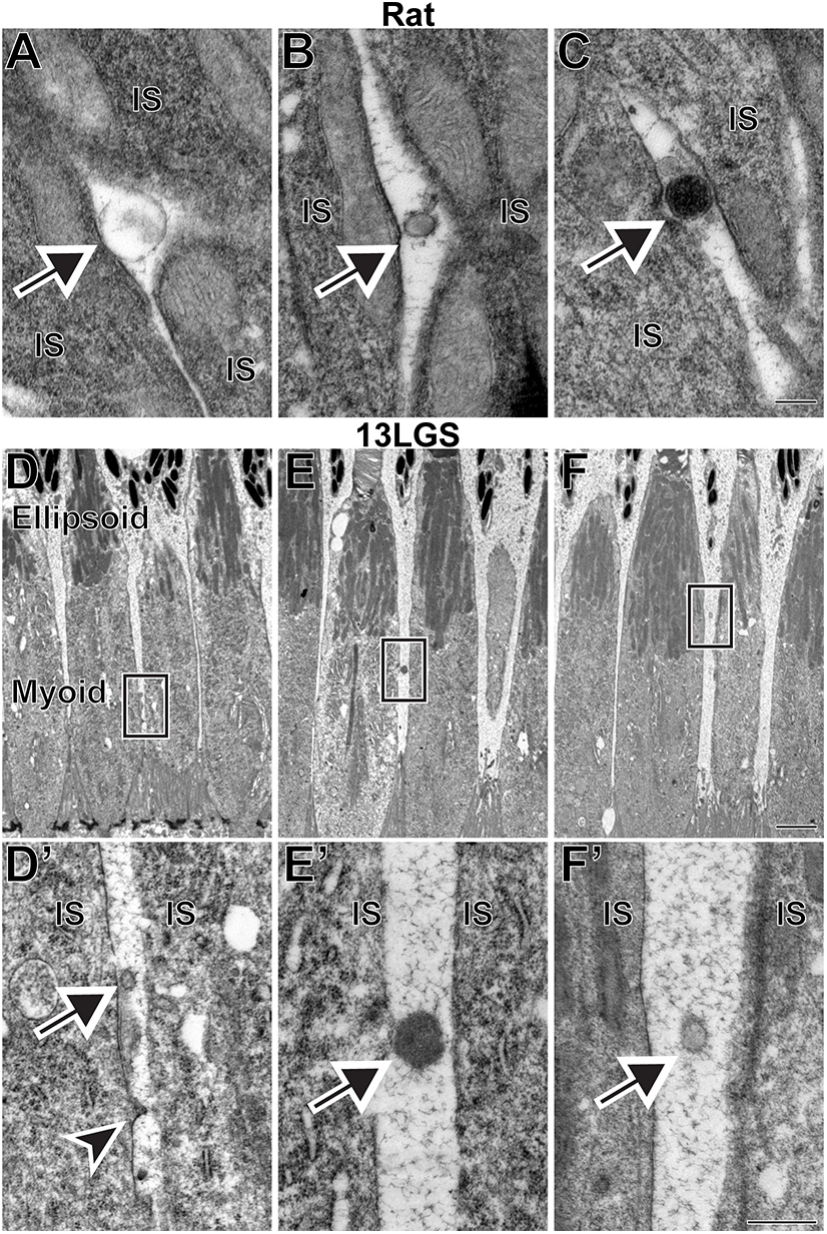
**Examples of microvesicles located next to the inner segments of WT rat and 13-lined ground squirrel (13LGS) photoreceptors.** (A-C) Microvesicles with a variety of staining patterns in TEM images of retinal sections from WT rats stained with tannic acid/uranyl acetate. (D,E,F) Low-magnification TEM images of WT 13LGS retinal sections stained with tannic acid/uranyl acetate. Boxed areas are magnified in D′,E′,F′. In all panels, arrows point to microvesicles; arrowhead points to a microvesicle budding off of a cone inner segment. IS, inner segment. Scale bars: 0.2 µm (A-C); 2 µm (D,E,F); 0.5 µm (D′,E′,F′). A total of four rats and four 13LGS were analyzed.

To identify whether microvesicle release from inner segments of healthy photoreceptors is a conserved phenomenon across mammalian species, we analyzed the retinas of WT rat and 13LGS using TEM. In the rat, we observed microvesicles surrounding photoreceptor inner segments comparable to those in mice, including a range of staining density patterns (Fig. 5A-C). We next analyzed retinas of the 13LGS, which is the most commonly used model of a cone-dominant mammalian species. The inner segments of 13LGS photoreceptors were highly compartmentalized, with mitochondria concentrating in a region known as the ellipsoid and other organelles residing primarily in the myoid (Fig. 5D-F′). We observed microvesicles adjacent to both the myoid (Fig. 5D-E′) and the ellipsoid (Fig. 5F,F′). On occasion, we observed microvesicles budding directly from cone inner segments (Fig. 5D,D′). Overall, our observations in multiple species suggest that microvesicle release is a conserved process occurring in healthy mammalian photoreceptors. The observations of microvesicles budding from rods and cones indicate that this process takes place in both types of photoreceptors.

### Microvesicles can be reabsorbed by photoreceptors

**Fig. 6.**
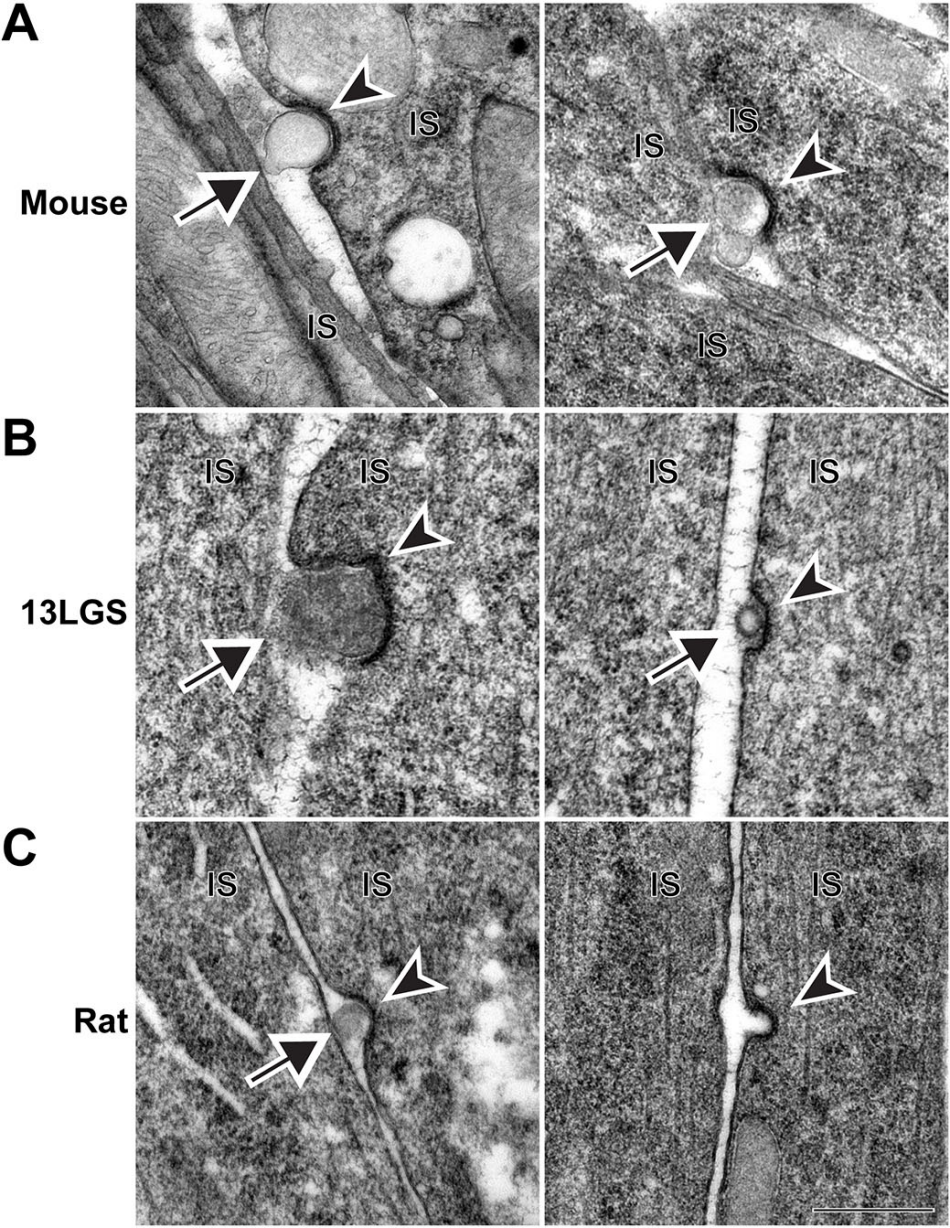
**Microvesicles can be endocytosed by the photoreceptor inner segment.** (A) TEM images of WT mouse retinal sections stained with tannic acid/uranyl acetate, which show microvesicles adjacent to endocytic, clathrin-coated pits. (B) TEM images of WT 13LGS retinal sections stained with tannic acid/uranyl acetate, which show microvesicles adjacent to endocytic, clathrin-coated pits. (C) TEM images of WT rat retinal sections stained with tannic acid/uranyl acetate. Endocytic, clathrin-coated pits are observed either with (left) or without (right) an adjacent microvesicle. In all panels, arrows point to microvesicles; arrowheads point to darkly stained clathrin-coated pits. IS, inner segment. Scale bar: 0.5 µm. A total of nine mice, four 13LGS and four rats were analyzed.

During the course of these studies, we observed a very small fraction (<2%) of microvesicles that were in the process of being endocytosed by photoreceptor inner segments (Fig. 6). These microvesicles were associated with electron-dense structures with an appearance of classical clathrin-coated endocytic pits. Although it has been previously shown that the inner segment is capable of endocytosis (Hollyfield and Rayborn, 1987), the scope and the functional role of this process have not been investigated. Our current observations suggest that at least a subset of these endocytic events involves the reabsorption of microvesicles, perhaps leading to their final degradation. However, the majority of endocytic pits that we observed were not associated with microvesicles, indicating that the scope of inner segment endocytosis must extend beyond this role.

### Some microvesicles contain rhodopsin

SeveralFig. S2 studies performed with mutant mouse models used immunogold labeling to demonstrate that the extracellular vesicles surrounding photoreceptor inner segments contain the visual pigment rhodopsin (Blanks and Spee, 1992; Li et al., 1996; Pazour et al., 2002; Lodowski et al., 2013). More recently, it has been suggested that the release of these vesicles in pathological conditions serves as a means of actively clearing rhodopsin mislocalized to the inner segment plasma membrane of these mutants (Lodowski et al., 2013). Interestingly, a minor level of rhodopsin mislocalization also occurs in WT photoreceptors (Tam et al., 2006; Chadha et al., 2019). Therefore, we hypothesized that the same mechanism of rhodopsin clearance may function in normal rods and cones.

**Fig. 7.**
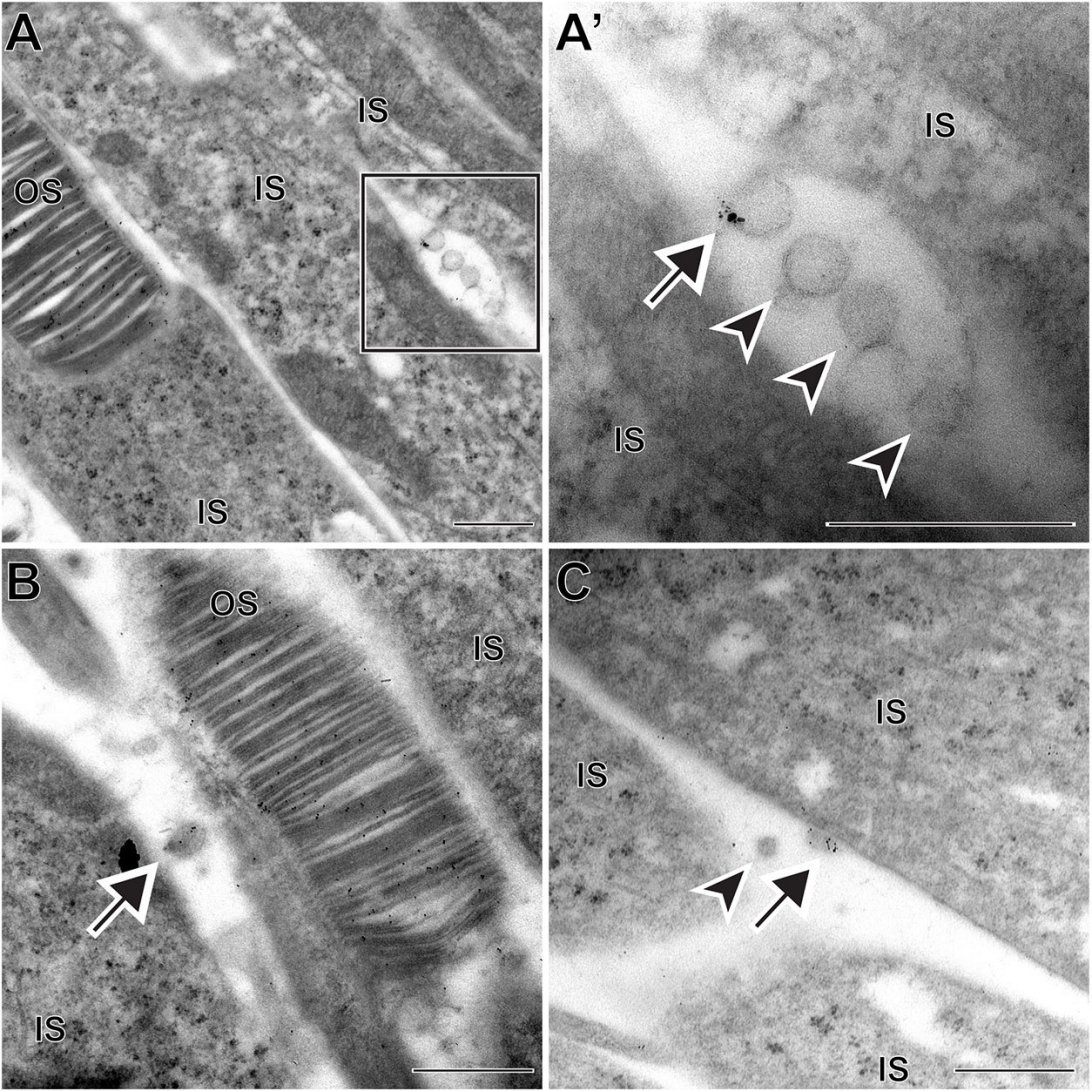
**A subset of microvesicles contain rhodopsin.** (A,B) TEM images from immunogold labeling of WT mouse retina using the 4D2 anti-rhodopsin antibody. Boxed area is magnified in A′. The majority of gold particles label the outer segment; microvesicles can be either labeled or unlabeled. (C) An example of a microvesicle labeled with the 1D4 anti-rhodopsin antibody. In all panels, arrows point to microvesicles labeled with anti-rhodopsin antibodies; arrowheads point to unlabeled microvesicles. Note that the granular structure of inner segment ribosomes can be particularly well distinguished from the gold particles in higher-magnification images. IS, inner segment; OS, outer segment. Scale bars: 0.5 µm. A total of 37 microvesicles were analyzed, of which 14 were labeled with anti-rhodopsin antibodies.

To test this hypothesis, we conducted immunogold labeling of rhodopsin in retinal sections of WT mice (Fig. 7). Indeed, we observed that ∼40% of the microvesicles were labeled with multiple gold particles, whereas labeling of the adjacent inner segment plasma membrane was sparse. This result is consistent with the mechanism whereby occasionally mislocalized rhodopsin molecules can be extruded from the inner segment plasma membrane within microvesicles.

We should mention that rhodopsin-positive extracellular vesicles have previously been observed *in vitro* in cultured frog retinas (Besharse and Wetzel, 1995). At the time, the authors hypothesized that these vesicles could be involved in the transport of rhodopsin from the inner to outer segment. However, it is currently accepted that rhodopsin is delivered to the outer segment through the connecting cilium (e.g. Chadha et al., 2019), which makes it possible that the extracellular vesicles observed by Besharse and Wetzel (1995) could be related to those observed in the current study.

**Fig. 8.**
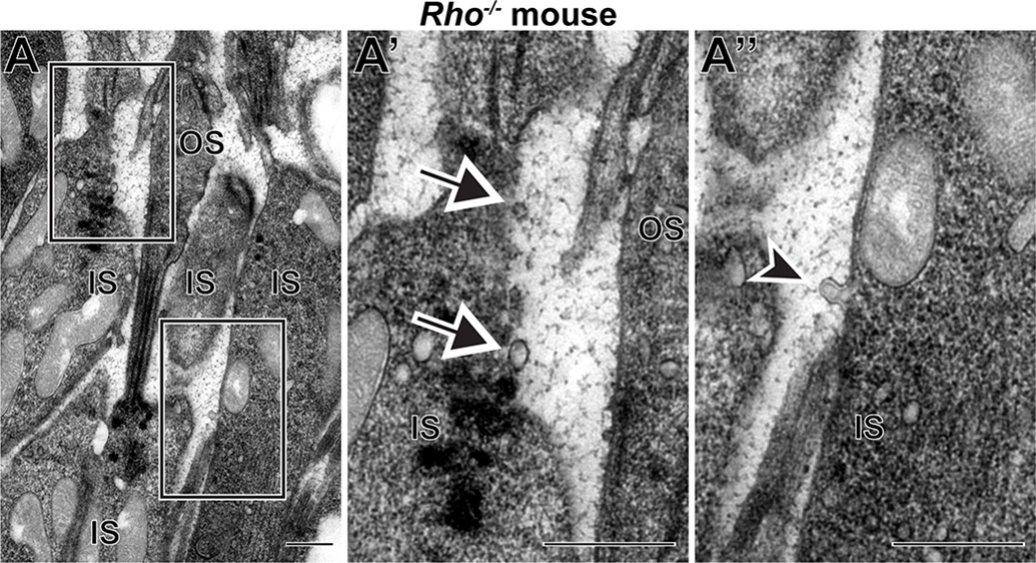
**Rhodopsin is not required for microvesicle release from rod inner segments.** Representative TEM image of a retinal section from a *Rho^−/−^* mouse stained with tannic acid/uranyl acetate. Boxed areas in A are magnified in A′ and A″. Arrows point to microvesicles; arrowhead points to a microvesicle in the process of budding from the inner segment. IS, inner segment. OS, outer segment. Scale bars: 0.5 µm. A total of four *Rho^−/−^* mice were analyzed.

Yet, over half of the microvesicles were not labeled for rhodopsin, suggesting that they contain alternative cargo. To further investigate whether microvesicle release can occur independently of rhodopsin, we analyzed the retinas of rhodopsin knockout (*Rho^−/−^*) mice at an early age preceding major photoreceptor cell death in this model. We observed microvesicles located next to photoreceptor inner segments, including an example of a vesicle budding directly off the plasma membrane and not connected to a mitochondrion (Fig. 8). These data demonstrate that microvesicle release from the inner segment plasma membrane does not require rhodopsin and, therefore, these vesicles contain alternative cargo.

## DISCUSSION

An abundant accumulation of extracellular vesicles around photoreceptor inner segments has been observed in multiple animal models of retinal degeneration, particularly those associated with defects in intracellular protein trafficking. We now demonstrate that the release of microvesicles from the inner segment is an intrinsic ability of healthy mammalian photoreceptors. This suggests that microvesicle release represents a homeostatic mechanism responsible for the removal of mislocalized or damaged proteins from the cell.

One important issue to consider is whether the vesicles observed in this study are present *in vivo* or they represent artifacts of tissue processing. For example, the membranes of newly forming photoreceptor discs at the base of the outer segment vesiculate during imperfect tissue fixation (for a detailed discussion, see Ding et al., 2015). Although we cannot prove that every single vesicular structure observed in this study was present in the retina prior to tissue fixation, there are multiple arguments to support the existence of these microvesicles in live retinas. First, we only analyzed retinas in which the nascent discs at the base of rod outer segments (which are extremely susceptible to vesiculation during fixation) exhibited normal structure. Second, the frequency at which we observed microvesicles was incredibly consistent among individual animals and animal ages. Third, the documented artifacts of tissue vesiculation consist of fragmentation of elaborate membrane structures into less complex vesicular structures (Ding et al., 2015). This is inconsistent with an artificial origin of highly elaborate structures, observed in this and previous studies, that originate at the mitochondria, trespass the plasma membrane and expand outside the cell. Fourth, a subset of microvesicles were endocytosed by clathrin-coated pits, which could not be an artifact of tissue fixation. Fifth, an increased accumulation of extracellular vesicles surrounding inner segments has been observed in a wide variety of mutant and transgenic animals, supporting the notion that microvesicle release is a real phenomenon taking place at different levels in healthy and degenerative conditions.

Regarding the composition of microvesicles released from WT photoreceptors, our study revealed that their cargo is variable. A fraction of microvesicles contains rhodopsin, which mirrors the findings in pathological models. As noted above, we speculate that release of rhodopsin-containing vesicles serves to dispose a small fraction of rhodopsin naturally mislocalizing to the inner segment. Why would such a mechanism exist in addition to the classical endolysosomal pathway? One possible explanation can be found in two studies of transgenic frogs expressing mutant rhodopsin (Ropelewski and Imanishi, 2019, 2020). The authors noted that both microvesicle release and endolysosomal degradation are capable of removing mutant rhodopsin from the inner segment plasma membrane. However, the endolysosomal pathway appears to be less specific because it removed the resident inner segment protein Na^+^/K^+^-ATPase along with rhodopsin, whereas no Na^+^/K^+^-ATPase was present in microvesicles containing mutant rhodopsin. Therefore, a potential advantage of the microvesicle release mechanism may relate to cargo selection specificity.

We also observed a subset of budding vesicles that were interconnected with inner segment mitochondria. Together with two recent studies describing possibly related structures in WT fish cones (Giarmarco et al., 2020) and primate photoreceptors (Hayes et al., 2021), these observations suggest that photoreceptor mitochondria have the ability to extrude some of their material to the extracellular space. Such a release of mitochondrial material has been observed in other cell types (Simpson and Kling, 1968; Gasko and Danon, 1972), and is thought to take place under both normal and pathological conditions (Lyamzaev et al., 2022). However, three questions regarding the mitochondria-associated structures in the photoreceptor inner segment remain to be addressed. First, are the extracellular portions of these structures ever released as vesicles? Second, if they are, then what fraction of the extracellular vesicles do they represent? Third, what is the exact content of these vesicles? Seeking answers to these questions is the challenge of future experiments that will elucidate the functional significance of this phenomenon.

Whereas the disposal of unwanted material from the cell is the most likely functional role of these vesicles, it is tempting to speculate that microvesicles could also be involved in the transfer of cytoplasmic material, such as proteins or RNA, between cells. The phenomenon of ‘material transfer’ has been previously reported to occur between endogenous and transplanted photoreceptors (Pearson et al., 2016; Santos-Ferreira et al., 2016; Singh et al., 2016; Ortin-Martinez et al., 2017). Although it has been hypothesized that material transfer may occur through extracellular vesicles (Gasparini et al., 2019), two recent studies argue that cytoplasmic material is transferred through nanotubes rather than vesicles (Kalargyrou et al., 2021; Ortin-Martinez et al., 2021). Thus, a more comprehensive characterization of these microvesicles' composition is required to consider whether they participate in the exchange of cytoplasmic material between cells.

There are several possibilities regarding the ultimate fate of released microvesicles. Here, we showed that photoreceptor inner segments endocytose at least some of them, perhaps directing them to final degradation in the endolysosomal pathway. Another possibility is that a fraction of microvesicles are engulfed by Müller glial cells, given the proximity of their apical processes to photoreceptor inner segments. A not mutually exclusive alternative is that some vesicles are engulfed by the retinal pigment epithelium (RPE), as suggested in the case of mutant rhodopsin (Ropelewski and Imanishi, 2020). However, the site of vesicle release in the inner segment of WT photoreceptors is separated from the RPE by tightly packed outer segments. This argues against a major RPE involvement in normal retinas, although the RPE could be heavily involved when outer segment integrity is distorted in pathology. Another major player under pathological conditions may be activated microglia, which have been shown to engulf outer segment-derived vesicles (Spencer et al., 2019). However, any involvement of microglia in microvesicle clearance in WT retinas is highly unlikely given their localization to the synaptic layers.

The goal of future studies is to define the overall impact of microvesicle release on maintaining the healthy status of normal photoreceptor cells. This would require elucidating the mechanism underlying microvesicle release and understanding how it could be manipulated. A related question is whether the massive microvesicle release observed in mutant photoreceptors ameliorates or exacerbates the ongoing pathology. It is intriguing to speculate that modulating this pathway in a desired direction could serve as a potential therapeutic approach for treating a group of retinal degenerative diseases.

## MATERIALS AND METHODS

### Animal husbandry

Animal maintenance and experiments were approved by the Duke Institutional Animal Care and Use Committee (Durham, NC, USA) and the University of Wisconsin Oshkosh Institutional Animal Care and Use Committee (Oshkosh, WI, USA). WT mice (*Mus musculus*) were C57BL/6J (The Jackson Laboratory stock #000664). *pcd^5J^* mice (described in Chakrabarti et al., 2006; The Jackson Laboratory stock #004518) were obtained from Dr Albert La Spada (University of California Irvine, Irvine, CA, USA). *Rho^−/−^* mice are described in Lem et al. (1999). All mice were backcrossed to the C57BL/6J background. Mice were genotyped to ensure that they did not contain either the *rd8* (*Crb1*) (Mattapallil et al., 2012) or *rd1* (*Pde6b*) (Pittler et al., 1993) mutations commonly found in inbred mouse strains. Long-Evans rats (*Rattus norvegicus*) were obtained from Charles River Laboratories (stock #006). 13LGS (*Ictidomys tridecemlineatus*) were maintained in a colony at the University of Wisconsin-Oshkosh. The ages of WT animals ranged between P30 and P180 for mice and P30 and P60 for rats. 13LGS were live captured from the wild with estimated ages ranging from 2 to 4 years. They were analyzed in a non-hibernating state. All experiments were performed with animals of randomized sex.

### Tissue processing

Tissue fixation was performed as described previously (Ding et al., 2015). In the morning after lights were turned on, anesthetized animals were transcardially perfused with 2% paraformaldehyde, 2% glutaraldehyde and 0.05% calcium chloride in 50 mM MOPS (pH 7.4) resulting in exsanguination. Enucleated eyes were fixed for an additional 2 h in the same fixation solution at room temperature prior to processing.

For samples contrasted with osmium tetroxide (Fig. 1), eyecups were dissected from fixed eyes and cut in half through the optic nerve. Tissue was treated with 2% osmium tetroxide (Electron Microscopy Sciences, Hatfield, PA, USA), gradually dehydrated with ethanol and infiltrated and embedded in Embed 812 resin (Electron Microscopy Sciences). For samples contrasted with tannic acid/uranyl acetate (Figs 2-6 and 8; Movies 1-4), eyecups were dissected from fixed eyes, embedded in 2.5% low-melt agarose (Precisionary, Greenville, NC, USA) and cut into 200 µm-thick slices on a Vibratome (VT1200S; Leica, Buffalo Grove, IL, USA). Agarose sections were treated with 1% tannic acid (Electron Microscopy Sciences) and 1% uranyl acetate (Electron Microscopy Sciences), gradually dehydrated with ethanol and infiltrated and embedded in Spurr's resin (Electron Microscopy Sciences).

### TEM

Sections (70 nm) of the central retina were cut from resin-embedded samples, placed on copper grids and counterstained with 2% uranyl acetate and 3.5% lead citrate (Ted Pella, Redding, CA, USA). The samples were imaged on a JEM-1400 electron microscope (JEOL, Peabody, MA, USA) at 60 kV with a digital camera (BioSprint; AMT, Woburn, MA, USA). Image analysis and processing were performed with ImageJ.

### 3D-ET

Either semi-thin (250 nm) or thick (750 nm) sections of the central retina were cut from resin-embedded samples and placed on 50 nm Luxel film slot grids. The grids were glow-discharged on both sides, and gold particles were deposited on the sample surfaces to serve as fiducial markers. For semi-thin sections, 5 nm gold particles were used; for thick sections, a mixture of 10 nm, 20 nm and 60 nm gold particles was used. 3D-ET was conducted on a Titan Halo (FEI, Hillsboro, OR, USA) operating at 300 kV in TEM mode for semi-thin sections or in STEM mode for thick sections. A four-tilt series data acquisition scheme previously described (Phan et al., 2017) was followed in which the specimen was tilted from −60° to +60° every 0.25° at four evenly distributed azimuthal angle positions. The micrographs were collected on a 8k×8k direct detector (DE64; Direct Electron, San Diego, CA, USA) in TEM mode or with a high-angle annular dark field (HAADF) detector in STEM mode. The final volumes were generated using an iterative reconstruction procedure (Phan et al., 2017). Manual segmentations and the corresponding animations were performed using 3dmod software (version 4.11; University of Colorado Boulder, Boulder, CO, USA).

### Immunogold labeling

Immunogold staining was performed as previously described (Ding et al., 2015). Eyes were fixed and cut into agarose sections as described above. Sections were treated with 0.5% tannic acid, cryoprotected with 30% glycerol in 0.1 M sodium acetate and freeze-substituted overnight in 4% uranyl acetate/95% methanol with gentle agitation in a dry ice/ethanol bath. Sections were then infiltrated with Lowicryl HM-20 (Electron Microscopy Sciences), slowly warmed to 4°C and polymerized for 3 days under UV light.

Sections (70 nm) of the central retina were cut from embedded samples, placed on nickel grids and treated with 10 mM citrate buffer (pH 6.0) containing 0.005% Tergitol NP-10 at 60°C. Grids were blocked with 1% glycine in Tris-buffered saline (pH 7.6) containing 0.005% Tergitol NP-10 and incubated overnight with either a 1:750 dilution of 1D4 (ab5417; Abcam, Cambridge, UK) or a 1:2000 dilution of 4D2 (ab98887; Abcam) primary mouse monoclonal antibody against rhodopsin. Grids were subsequently blocked with 1% donkey serum and incubated with donkey anti-mouse IgG conjugated with 6 nm colloidal gold (Jackson ImmunoResearch Laboratories, West Grove, PA, USA). Grids were counterstained with 2% uranyl acetate and 3.5% lead citrate (Ted Pella). Samples were imaged on a JEM-1400 electron microscope (JEOL) at 60 kV with a digital camera (BioSprint). Image analysis and processing was performed with ImageJ.

### Quantification of extracellular vesicle frequency

Using TEM of WT mouse retinal sections, we counted the number of extracellular vesicles adjacent to inner segments in a region containing 200 photoreceptors. This analysis was performed for three WT mice at three different ages: P30, P60 and P180. Values are reported as the frequency of extracellular vesicles observed per photoreceptor inner segment.

## Supplementary Material

10.1242/dmm.049871_sup1 Supplementary information
