# Supplementary material for: Microvesicle release from inner segments of healthy photoreceptors is a conserved phenomenon in mammalian species
Source: Dis Model Mech. 2022 Nov 23;15(11):dmm049871. doi: 10.1242/dmm.049871 (PMC9796728; doi:10.1242/dmm.049871)
Supplement: Supplementary information [file dmm-15-049871-s1.pdf]

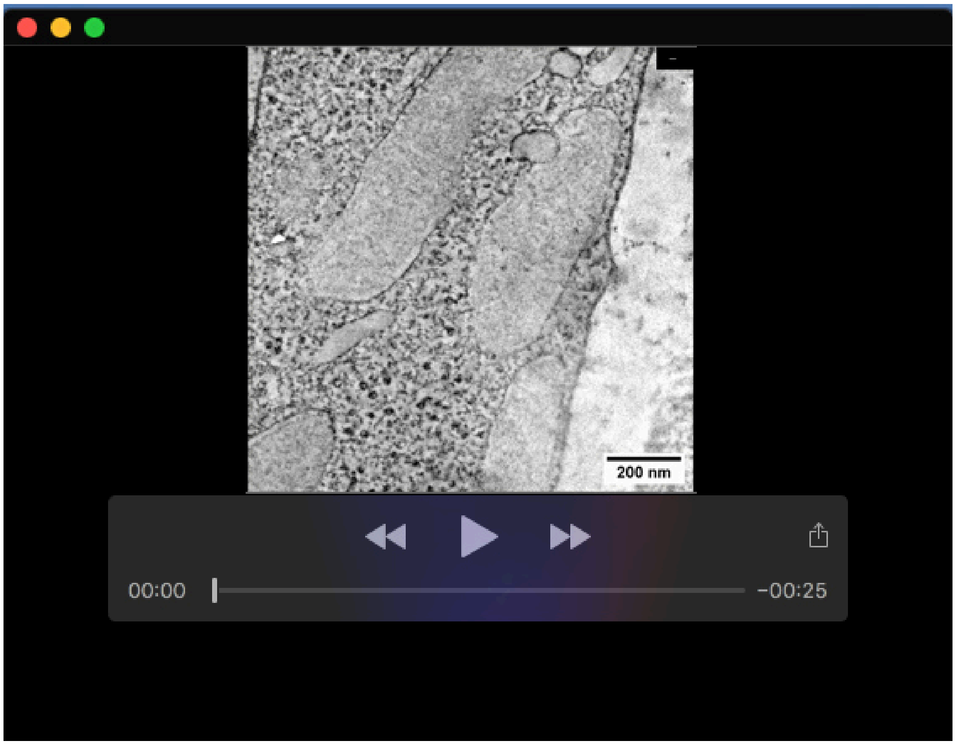

Movie 1. Microvesicle released from the inner segment plasma membrane with a staining pattern matching the cytoplasm. Tomogram associated with **Fig. 2A**.

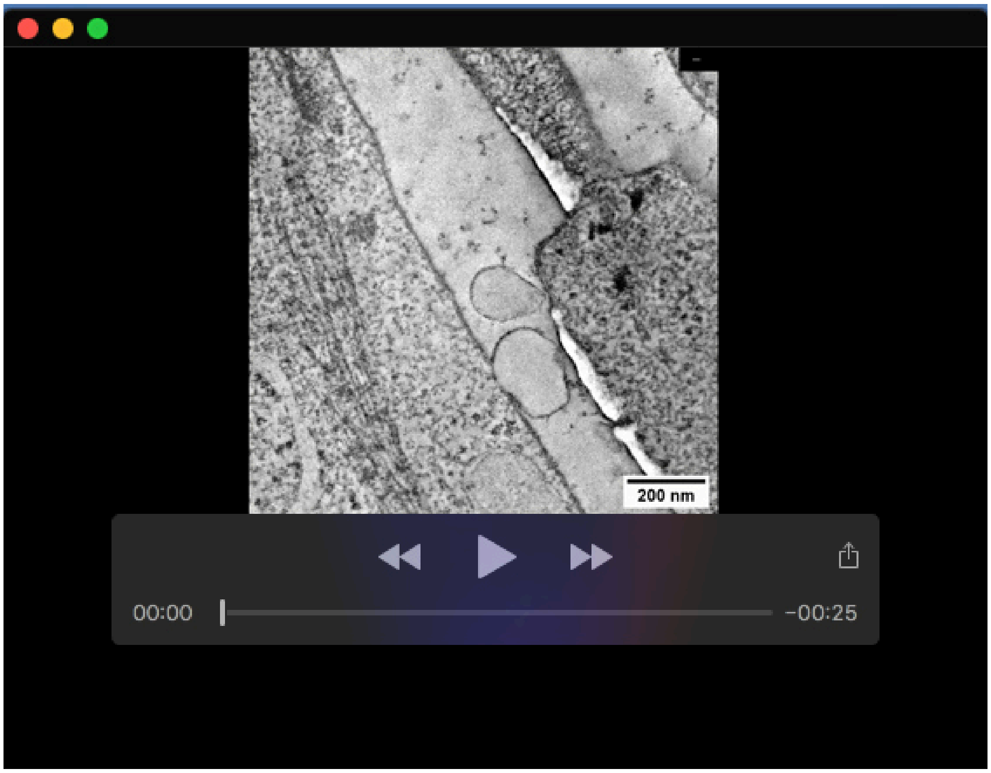

Movie 2. Microvesicle released from the inner segment plasma membrane with a clear staining pattern. Tomogram associated with **Fig. 2B**.

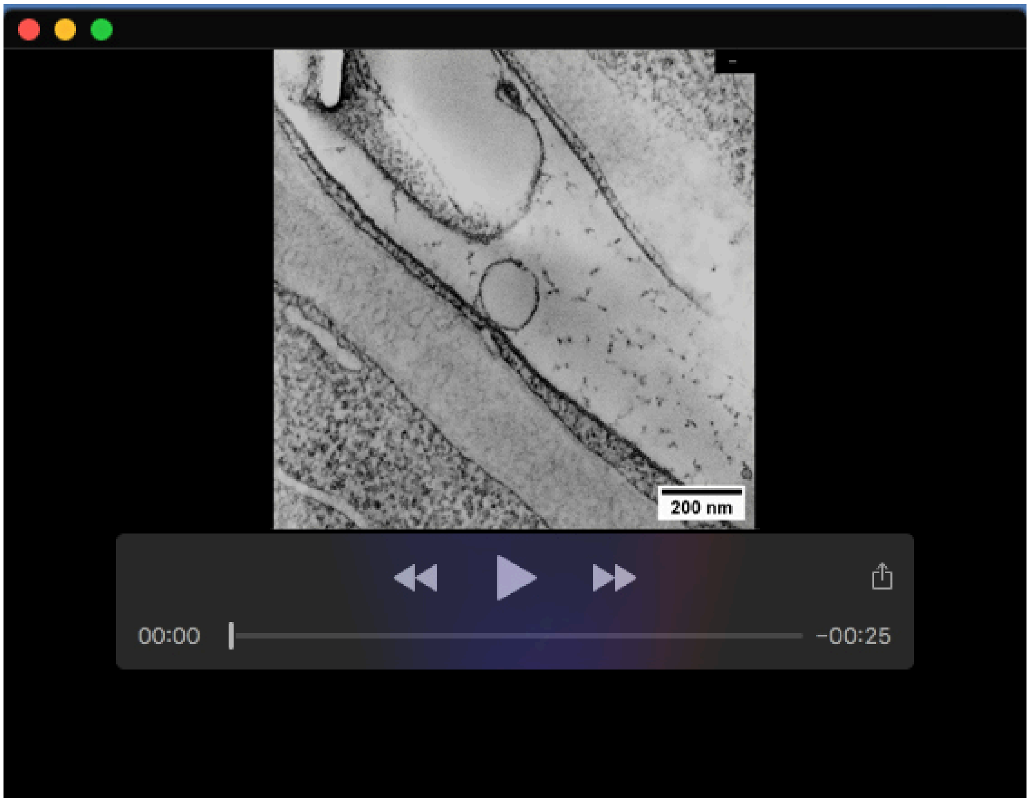

Movie 3. Extracellular vesicular structure connected to inner segment mitochondrion through a short membrane tunnel. Tomogram associated with **Fig. 3A**.

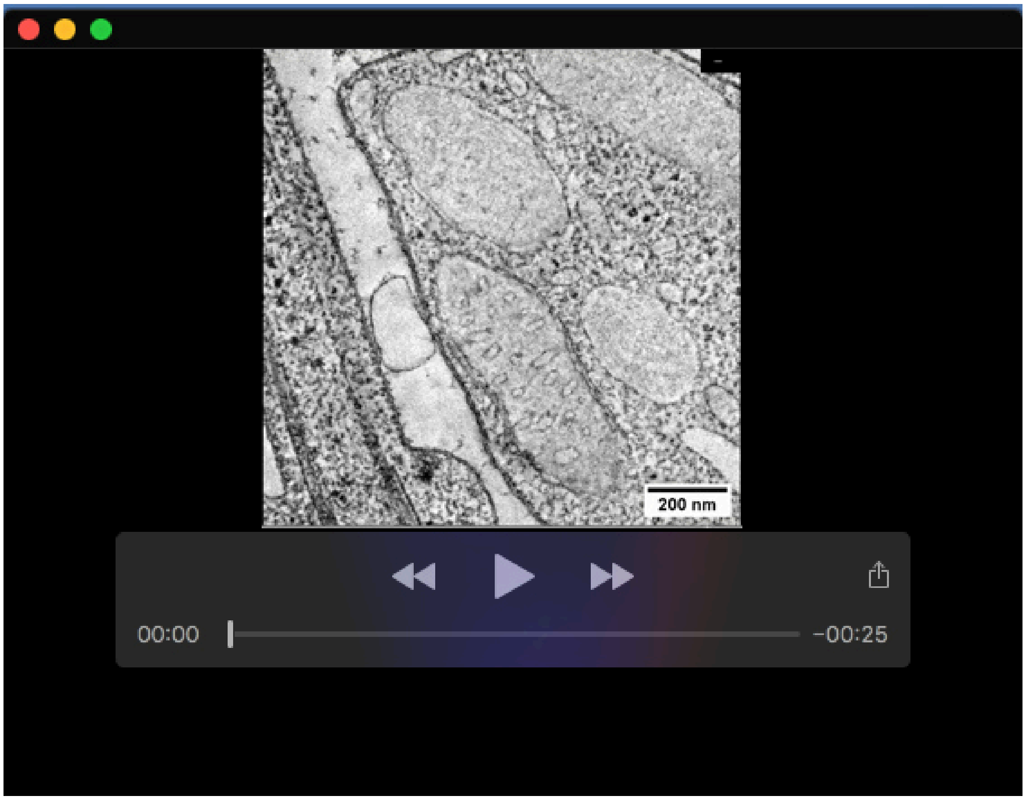

Movie 4. Extracellular vesicular structure connected to inner segment mitochondrion through a complex membrane tunnel. Tomogram associated with **Fig. 4A**.

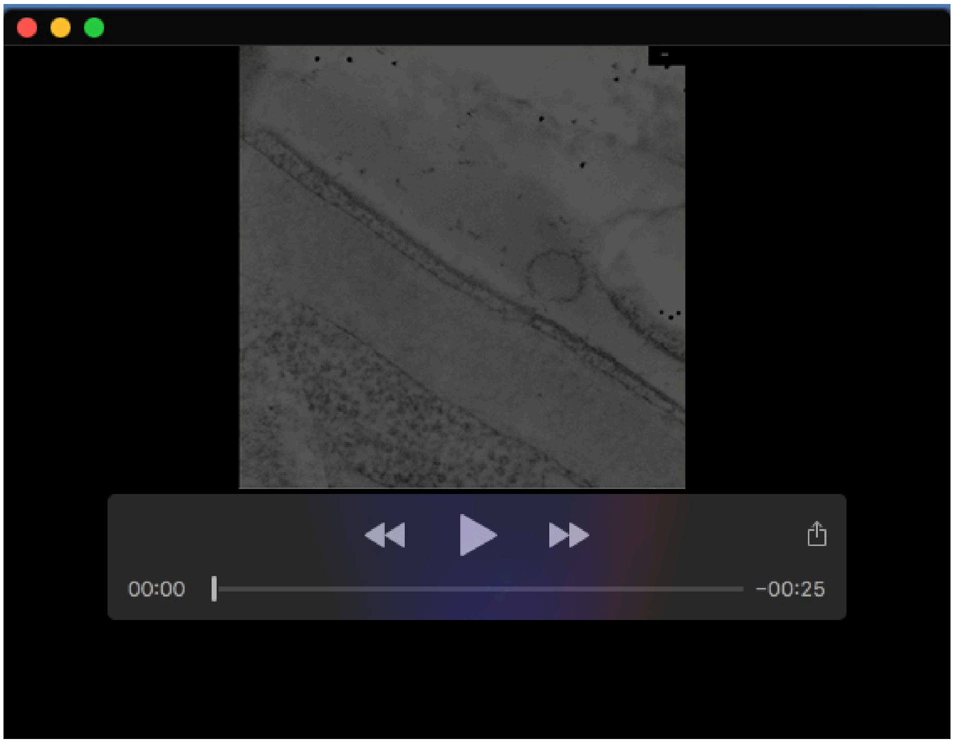

Movie 5. Segmentation of an extracellular vesicular structure connected to inner segment mitochondrion through a short membrane tunnel. Tomogram segmentation associated with **Fig. 3B**.

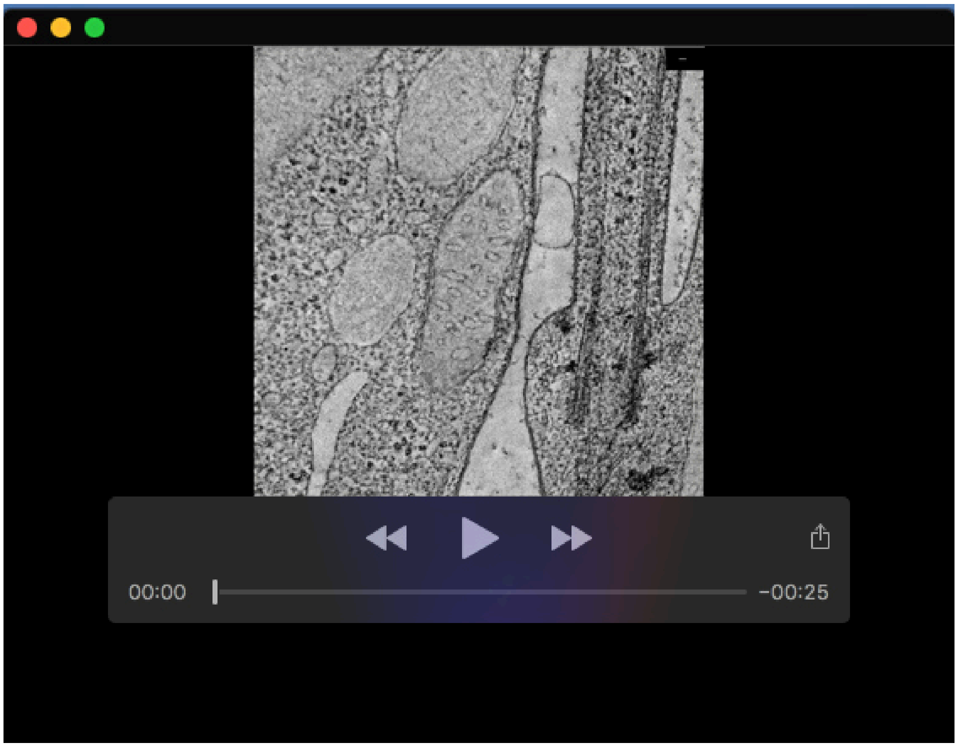

Movie 6. Segmentation of an extracellular vesicular structure connected to inner segment mitochondrion through a complex membrane tunnel. Tomogram segmentation associated with **Fig. 4B**.
